# Genetic features and therapeutic relevance of emergent circulating tumor DNA alterations in refractory non-colorectal gastrointestinal cancers

**DOI:** 10.1038/s41467-022-35144-1

**Published:** 2022-12-03

**Authors:** David Hsiehchen, Leslie Bucheit, Dong Yang, Muhammad Shaalan Beg, Mir Lim, Sunyoung S. Lee, Pashtoon Murtaza Kasi, Ahmed O. Kaseb, Hao Zhu

**Affiliations:** 1 Division of Hematology and Oncology, Department of Internal Medicine, University of Texas Southwestern Medical Center, Dallas, TX USA; 2 Guardant Health Inc, Redwood City, CA USA; 3 Department of Gastrointestinal Medical Oncology, University of Texas MD Anderson Cancer Center, Houston, TX USA; 4 Weill Cornell Medicine, Englander Institute of Precision Medicine, Meyer Cancer Center, New York, NY USA

**Keywords:** Bile duct cancer, Cancer therapeutic resistance, Pancreatic cancer, Hepatocellular carcinoma, Cancer genomics

## Abstract

Acquired resistance to systemic treatments is inevitable in most cancers, but the genetic basis for this in many cancer types has remained elusive due to constraints in obtaining tissue specimens longitudinally. In the management of gastrointestinal cancers, molecular profiling is conventionally performed at a single time point, although serial evaluations may yield biological insights that inform treatment decisions. We characterize genetic changes in serial liquid biopsies which provide real-time snapshots of tumor genetics and heterogeneity in refractory non-colorectal gastrointestinal cancers, and determine the clinical utility of repeat circulating tumor DNA (ctDNA) testing. In a national cohort of 449 patients with pancreatic, biliary, esophagogastric, and hepatocellular cancers, resistance to conventional therapies is broadly associated with tumor evolution. Emergent ctDNA alterations only detectable at progression occurs in 63% of patients and are frequently associated with treatment actionability. Tumor mutation burden is dynamic in cancers undergoing treatment, but is not associated with time to progression. Objective tumor responses in a case series of patients receiving treatment matched to emergent alterations show that repeat liquid biopsies may have clinical benefit by expanding treatment options in advanced gastrointestinal cancers.

## Abstract (web summary)

Acquired resistance can be associated with genetic changes, however, clinical molecular profiling is usually only considered at the time of diagnosis. Here, the authors use serial ctDNA profiling of 449 gastrointestinal cancers to demonstrate widespread tumour evolution associated with treatment resistance and its potential implications for treatment.

## Introduction

Current knowledge of cancer genomes has primarily stemmed from tissue-based analyses of cancers that have not been previously exposed to systemic treatments^1–4^. This coincides with the conventional practice of performing clinical sequencing on tissue specimens collected for diagnostic purposes prior to treatment. However, tumor heterogeneity and changes in clone architecture are hallmarks of acquired resistance to treatment, and these features cannot be inferred from molecular profiles at a single site or point in time^5–9^. Multi-site and longitudinal sequencing of tissue in select diseases and in small patient cohorts show that resistance to targeted therapies is associated with numerous mutation events which are often not observed in pre-treatment specimens from a single site^5,7,8,10^. Thus, the genetic basis of resistance for many treatments across most cancer types has not been resolved despite the abundance of cancer genomes sequenced to date.

Detection of circulating tumor DNA (ctDNA), also referred to as liquid biopsies, provides a non-invasive method of detecting tumor-associated molecular alterations. A minority of cancers have low rates of ctDNA shedding which may preclude detection, although this is generally associated with early-stage diseases^11^. Nonetheless, early investigations demonstrated the sensitivity and specificity of ctDNA profiling as a diagnostic molecular assay to guide treatment selection, particularly for targeted therapies^12,13^. Recent advances have broadened the potential utility of liquid biopsies into cancer prognostication, minimal residual disease detection, cancer screening, and pharmacodynamic monitoring^14–23^. Besides the greater convenience of liquid biopsies, ctDNA profiling offers notable advantages over tumor tissue analyses in characterizing intratumoral heterogeneity and evolutionary processes^12,13^. This can be attributed to the inability of a single tissue biopsy to comprehensively capture inter- and intra-lesional genetic heterogeneity^5^. Tumor-naive liquid biopsies are also not biased by discoveries in past tissue or liquid biopsies, and may thus capture unanticipated molecular events that are only enriched by the selective pressures of cancer therapies.

Liquid biopsies have been used to elucidate tumor cell evolution in several cancer types including non-small cell lung and colorectal cancer^5,24^. The role of ctDNA in tracking genetic changes in response to treatment has also been demonstrated across several studies in colorectal cancers^25–28^. Whether evolutionary changes in ctDNA, including the emergence of new alterations and changes in tumor mutation burden (TMB), have therapeutic relevance across cancer types remains undefined. Therefore, we analyze a national cohort of patients with advanced and refractory esophagogastric, pancreatic, biliary, and hepatocellular cancers who underwent serial liquid biopsies using a clinical ctDNA assay to characterize ctDNA changes over time. Our results uncover mechanisms of treatment resistance to conventional therapies and demonstrate a high prevalence of emergent ctDNA alterations at progression that are associated with treatment actionability. We also observe considerable fluctuations in TMB, and suggest the clinical utility of serial liquid biopsies in a case series of patients who received subsequent treatment matched to emergent alterations. As the current standard of practice is to obtain genomic profiling at a singular timepoint regardless of treatment history, our study supports the clinical utility of repeat tumor naive liquid biopsies in a subset of patients with advanced gastrointestinal cancers.

## Results

### Dynamic categories of ctDNA alterations

Our national cohort included 449 patients with advanced cancers who had baseline and post-progression liquid biopsies collected during the study period identified from a real-world genomic database including 146 with pancreatic adenocarcinoma carcinomas (PDAC), 134 intrahepatic cholangiocarcinomas (CCA), 133 esophagogastric carcinomas (EGC), and 36 hepatocellular carcinomas (HCC). Liquid biopsies were conducted using an FDA-approved gene-panel next-generation sequencing companion diagnostic assay (Guardant360, Guardant Health). Progression was defined by the treating physician based on evidence of radiographic progression consistent with RECIST 1.1.

As expected, among a subset of patients with serum tumor marker (CA 19-9 or AFP) assessed at the time of each liquid biopsy and accessible radiographic imaging, there was a positive correlation between percent change in ctDNA levels with serum tumor markers and tumor dimensions (Supplementary Figure 1). To delineate changes in ctDNA alterations at progression, ctDNA alterations were categorized according to their dynamics as emergent, increasing, stable, decreasing, or lost (see Methods). Across all patients, there were 1535 unique alterations including missense, inframe, truncating, splice site, copy number alterations (CNAs), promoter, and synonymous mutations (Supplemental Fig. 2). Missense mutations were the predominant type of alteration detected across dynamic categories regardless of histology (68.1% in PDAC, 56% in EGC, 63% in CCA, 55% in HCC), and EGCs were associated with a greater number of CNAs compared to other cancer types (3.1% in PDAC, 10.8% in EGC, 4.4% in CCA, 4.8% in HCC, Chi-square test *p*-value < 0.001) (Supplemental Fig. 2). In downstream analyses, synonymous and benign variants were excluded (see Methods).

**Fig. 1:**
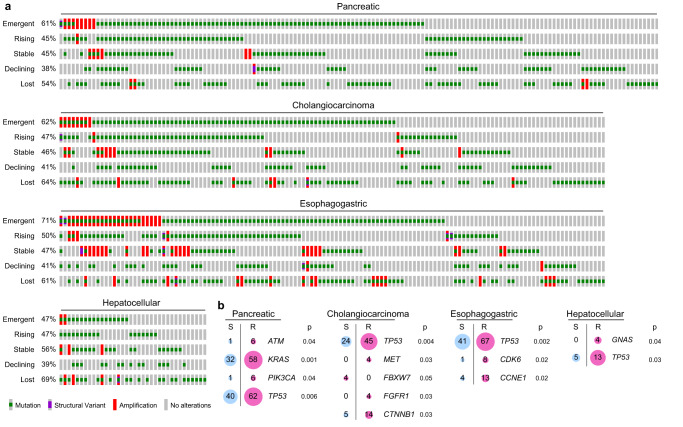
Dynamics in ctDNA alterations in refractory gastrointestinal cancers. **a** Oncoprint plots depict nonsynonymous ctDNA alterations categorized as either emergent, rising, stable, declining or lost across individual with gastrointestinal cancers. **b** Enrichment of resistance (R) or sensitive (S) alterations in genes with a significant association (*p* < 0.05) based on two-sided Fisher exact tests are shown. Pink and blue circles indicate the majority and minority of alterations found in each gene. For this exploratory analysis, no adjustments were made for multiple comparisons. Values in circles show the number of patients with ctDNA alterations.

Most refractory cancers demonstrated alterations that were emergent or had rising VAFs (Fig. 1a). Cancers with emergent mutations accounted for 61% of PDAC, 62% of CCA, 71% of EGC, and 47% of HCC cases (Fig. 1a). Few cancers had no detectable alterations at progression, with numbers ranging from 5.4% in PDAC to 13.8% for HCC (Fig. 1a). Among patients with no detectable alterations at baseline, emergent alterations were subsequently detected in 9 patients (6.1%) with PDAC, 10 patients (7.5%) with CCA, 12 patients (9%) with ESC, and 0 patients with HCC (Fig. 1a). A gene-level analysis showed that mutations in *TP53*, *KRAS*, *EGFR*, and *ATM* were among the most common emergent alteration in all cancer types (Supplemental Fig. 3). Other common emergent genes included histology-specific driver genes such as *BRCA2* and *CDKN2A* alterations in PDAC, *FGFR2* and *ARID1A* alterations in CCA, and *MET* and *CCNE1* alterations in EGC (Supplemental Fig. 3).

Low rates of ctDNA shedding as a consequence of limited tumor cell turn over or smaller tumor burden at baseline may contribute to an initial lack of detectable driver mutations. To assess if this factor may confound our findings, we analyzed patients with PDAC who had detectable *KRAS* mutations at progression but not at baseline. The rationale for studying this subset of patients is because *KRAS* mutations are pervasive in PDAC, suspected to be clonal in most cancers, and have a high-penetrance phenotype^29^. Notably, 13 of 16 PDAC cases with emergent *KRAS* mutations had other driver alterations detected at baseline (the remaining 3 cases had detectable synonymous mutations at baseline) and these baseline driver mutations were associated with higher variant allele frequencies (VAF) than those of emergent *KRAS* mutations (Supplemental Fig. 4). Thus, the inability to initially detect driver gene alterations in ctDNA may result from subclonal expansion rather than low-ctDNA shedding tumors.

### Treatment-resistant alterations inferred from ctDNA dynamics

ctDNA dynamics may be used to infer alterations associated with treatment resistance as changes in the detection and VAF of alterations can serve as pharmacodynamic readouts. We classified emergent, increasing, and stable pathogenic mutations as resistance alterations, and decreasing and lost pathogenic mutations as sensitive alterations. To determine whether a gene was associated with resistance or sensitivity to treatment, we compared the frequency of resistance and sensitive alterations in the same gene. Across all cancer types, *TP53* mutations were more frequently associated with resistance (Fig. 1b). In PDAC, *ATM*, *KRAS*, and *PIK3CA* mutations were also significantly associated with resistance (Fig. 1b). In CCA, *CTNNB1*, *MET*, and *FGFR1* mutations were associated with resistance while *FBXW7* mutations were associated with sensitivity (Fig. 1b). *CCNE1* and *CDK6* mutations were associated with resistance in EGC while *GNAS* mutations were associated with resistance in HCC (Fig. 1b). *FGFR2* mutations were not significant enriched among resistant alterations in CCA, but we observed two patients with *FGFR2* fusion CCA with emergent polyclonal *FGFR2* gatekeeper mutations, consistent with acquired resistance to ATP-competitive FGFR inhibitors (Supplemental Fig. 5)^28^.

### Characteristics of emergent ctDNA alterations

**Fig. 2:**
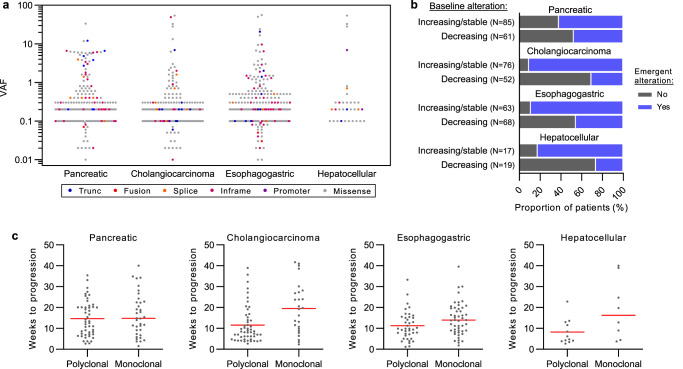
Characteristics of emergent ctDNA alterations. **a** VAF of emergent ctDNA alterations color-coded by mutation type are show in a dot plot. **b** Differences in proportions were assessed using the Chi-square test. Emergent ctDNA alterations were more prevalent in patients with increasing or stable maximal VAF in baseline alterations in PDAC (*p* = 0.04), CCA (*p* < 0.001), EGC (*p* < 0.001), and HCC (*p* < 0.001). Patients without any detectable baseline alterations were excluded. **c** Time to progression was shorter among cancers with multiple emergent alterations (polyclonal) versus single emergent alterations (monoclonal) in CCA (*P* = 0.002), EGC (*P* = 0.03), and HCC (*P* = 0.08) but not PDAC (*P* = 0.52).

We characterized the clinical features of emergent ctDNA alterations in the national cohort because these mutations may define new therapeutic targets for individual patients. Most emergent alterations had a VAF greater than 0.1% (92.2% in PDAC, 91.7% in CCA, 90.4% in EGC, and 100% in HCC), with the limit of detection being a VAF of 0.01% in the liquid biopsy assay (Fig. 2a). In most patients, the ratio of the maximal VAF of emergent alterations to the maximal VAF of baseline alterations was at least 0.1 (77.6% in PDAC, 59.7% in CCA, 56.3% in EGC, and 61.1% in HCC) although a subset of cases had VAF ratios greater than 1 (31.3% in PDAC, 11.9% in CCA, 20.3 in EGC, and 16.7% in HCC) (Supplemental Figure 6a). Patients with increasing or stable maximal VAF of any baseline alteration, likely denoting patients with an overall increase in tumor burden, were more commonly associated with obtaining emergent alterations (Fig. 2b). Nonetheless, 47.6% of PDAC, 30.8% of CCA, 45.6% of EGC, and 26.3% of HCC cases with a decline in the maximal VAF among baseline mutations, likely denoting patients with progressive disease such as new lesions with an overall decrease in tumor burden, had emergent alterations at progression (Fig. 2b). The maximal VAF at baseline was greater among cases with emergent alterations among CCA but not PDAC, EGC, or HCC, suggesting that baseline VAF may not be predictive of emergent alterations across cancer types (Supplemental Figure 6b). Collectively, these findings indicate that emergent alterations are readily detectable at progression, likely represent substantial expansions of subclones, and are observed in many patients regardless of VAF trends in baseline alterations.

The mean time until progression was 14.1- and 14.4 weeks among cases with and without emergent ctDNA alterations, respectively (Supplementary Figure 7). No differences in time to progression was noted within cancer types with the exception of EGC where emergent cases had shorter time to progression (13.7 weeks versus 17.8 weeks, *t* test *p* value = 0.03). However, emergent alterations were noted as soon as 3 to 4 weeks in multiple patients with early evidence of progression regardless of cancer type (Supplementary Figure 7). There was no significant correlation between the maximal VAF of emergent alterations and time to progression in any cancer type (Supplementary Figure 8a). There were also no statistical differences in time to progression between patients with rising maximal VAFs of baseline alterations but without emergent alterations, and patients with emergent alterations plus declining maximal VAFs of baseline alterations (Supplementary Figure 8b). However, patients with multiple emergent alterations likely denoting polyclonal resistance mechanisms had shorter time to progression than patients with a single emergent alteration in CCA, EGC, and HCC (Fig. 2c).

Driver fusion genes are uncommon but are frequently targetable alterations. We observed 3 emergent in-frame fusion ctDNA alterations including a *SATB1*-*RET* fusion at a VAF of 0.07% in a PDAC case with a maximal VAF in baseline mutations of 0.1% (Supplemental Figure 9). Breakpoints occurred in intron 11, a hotspot intron, of the *RET* gene, and intron 7 of the *SATB1* gene resulting in retention of an intact RET kinase domain in the fusion product (Supplemental Figure 9). In a patient with EGC, we observed a *CAPZA2*-*MET* fusion at a VAF of 0.05% in a patient with a maximal VAF in baseline mutations of 6.6% (Supplemental Figure 9). Breakpoints occurred in intron 7 of the *CAPZA2* gene, and exon 9 of the *MET* gene resulting in retention of an intact MET kinase domain in the fusion product (Supplemental Figure 9). In a separate patient with EGC, we observed an *FGFR2*-*WDR11*-*AS1* fusion at a VAF of 0.03% in a patient with a maximal VAF in baseline mutations of 0.1% (Supplemental Figure 9). Breakpoints occurred in exon 18 of the *FGFR2* gene, and intron 3 of the *WDR11-AS1* gene resulting in a 5’ kinase fusion product (Supplemental Figure 9).

A subset of patients in the national cohort had clinical sequencing performed on baseline single-site tissue specimens collected within 3 months of the baseline ctDNA specimen, which showed that 70.1% of mutations overlapped in baseline tissue specimens and baseline ctDNA (Supplemental Figure 10). However, only 6 of 16 patients had perfect concordance of all alterations in baseline tissue and ctDNA specimens, with 3 patients having alterations in baseline tissue that were not found in baseline ctDNA, while 9 patients having alterations in baseline ctDNA that were not found in baseline tissue (Supplemental Figure 10). Among the 13 patients in this patient subset with emergent ctDNA alterations, 2 patients had emergent ctDNA alterations identified in baseline tissue, but 12 patients also had emergent alterations not detected in baseline tissue. Collectively, these findings indicate that most alterations are concordant between baseline tissue and baseline ctDNA specimens, but many baselines and emergent ctDNA alterations are not detected by single-site biopsy sequencing.

### Evolution of TMB

**Fig. 3:**
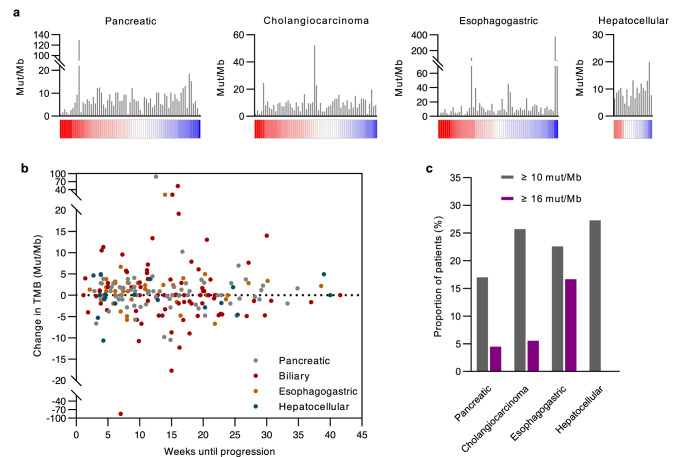
Evolution of tumor mutation burden. **a** Column charts depict baseline TMB values on the y-axis with patients on the *x* axis ranked by the percent change in TMB at progression. Patients with the greatest changes in TMB are in the far left of each column chart. Heatmaps along the *x* axis depict relative changes in TMB at progression calculated as the precent of TMB at baseline, with red indicating values of at least 200% and blue 0%. **b** Scatter plot shows relationship between absolute change in TMB and time to progression. **c** Column charts indicate the proportion of patients with a baseline TMB less than 10 or 16 mut/Mb who had a TMB at progression of at least 10 or 16 mut/Mb.

A proportion of patients in the national cohort had TMB determined through liquid biopsies at baseline and progression, including 69 patients with PDAC, 60 patients with CCA, 59 patients with EGC, and 19 patients with HCC. Tissue TMB data was available in a subset of patients which showed a positive and significant correlation between baseline ctDNA and tissue-derived TMB (Supplemental Figure 11a). Mean TMBs at baseline and progression were not significantly different (Supplemental Figure 11b). TMB at baseline and progression were also significantly correlated across all cancer types (Supplemental Figure 12). However, the explained variance of TMB at progression by TMB at baseline was moderate or low with coefficients of determination ranging from 76% for CCA to 18% with HCC, indicating considerable variations in TMB within the same patient (Supplemental Figure 12). A majority of cancers (60.9% in PDAC, 68.3 in CCA, 54.2% in EGC, and 52.6% in HCC) had an increase in TMB at progression, and there was no association between percent change in TMB and baseline TMB values (PDAC: *r* = 0.04, *p* value = 0.77; CCA: *r* = −0.19, *p* value = 0.15; EGC: *r* = −0.13, *p* value = 0.32; HCC: *r* = −0.36, *p* value = 0.13) (Fig. 3a). Prior studies show that TMB increases with age in several cancer types, but we found no correlation between the absolute change in TMB at progression and time to progression (PDAC: *r*-0.01, *p* value = 0.91; CCA: *r* = −0.14, *p* value = 0.21; EGC: *r* = 0.04, *p* value = 0.75; HCC: *r* = 0.15, *p* value = 0.54) (Fig. 3b)^30^. One patient with PDAC had a microsatellite instable cancer which was detected at both baseline and progression.

The FDA has approved a TMB cutoff of 10 mut/Mb to determine eligibility for immune checkpoint inhibitors in treatment-refractory solid cancers not otherwise eligible for immunotherapies^31^. Given that many cancers exhibited TMB changes in response to treatment, we assessed the proportion of cancers with a TMB <10 mut/Mb at baseline who subsequently had a TMB of at least 10 mut/MB at progression. Across all cancer types, there was a sizable minority of such cases who had a TMB at progression reaching the cutoff ranging from 17% in PDAC to 27.3% in HCC (Fig. 3c). Using a more stringent TMB cutoff of 16 mut/Mb, a value associated with clinical benefit from immune checkpoint inhibitors in lung cancers who had TMB determined from a liquid biopsy, showed a smaller fraction of patients who would have been potentially eligible for immune checkpoint inhibitors (Fig. 3c)^32^. In PDAC, CCA, and EGC, a large proportion of patients with a TMB of at least 10 at baseline who subsequently had a TMB of at least 10 mut/MB at progression was also observed ranging from 50% in PDAC to 78.6% in EGC using a 10 mut/Mb cutoff (Supplemental Figure 13). Similar results were observed when the analysis was repeated using the 16 mut/Mb cutoff (Supplemental Figure 13). In HCC, 50% of patients with a baseline TMB of at least 10 mut/Mb reached a cutoff of 10 mut/Mb at progression, and no patient had a TMB of at least 16 mut/Mb at progression (Supplemental Figure 13).

### Therapeutic relevance of emergent ctDNA alterations: molecular tumor board

**Fig. 4:**
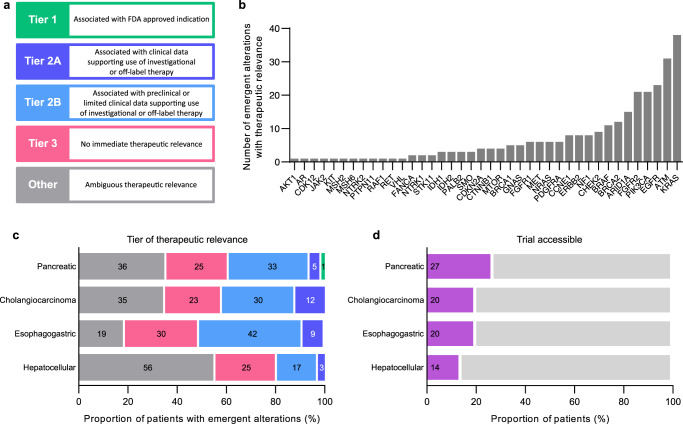
Therapeutic relevance of emergent ctDNA alterations. **a** Tiers of evidence used by the molecular tumor board to classify emergent ctDNA alterations by therapeutic relevance. **b** Distribution of therapeutically relevant alterations across genes. **c** Patients with emergent ctDNA alterations were categorized by the highest tier of evidence for any alteration. **d** Proportion of all patients in each cancer type who were eligible for an active clinical trial in the same state at the time of progression based on an emergent biomarker.

An interdisciplinary molecular tumor board comprised of oncologists, geneticists, pathologists, and pharmacists at a single institution systematically evaluated the clinical utility of emergent ctDNA alterations in the national cohort to guide treatment decisions (see Methods). Emergent alterations were categorized by tumor-specific tiers of evidence and potential of actionability based on similar classification schemes in prior studies^1,33,34^. Tier 1 and 2 alterations were considered to be clinically actionable using approved or investigational drugs, Tier 3 alterations were considered to be therapeutic targets not currently actionable, and Other alterations had ambiguous therapeutic relevance (Fig. 4a). A slight majority of emergent alterations were classified in Tier 1–3 across cancer types (Supplementary Figure 14). Five emergent alterations in PDAC and 0 alterations in other cancer types were classified as Tier 1. Tier 2A and 2B alterations accounted for 26.3%, 37.6%, 29%, and 17.9% of emergent alterations in PDAC, CCA, EGC, and HCC, respectively (Supplementary Figure 14). Tier 1–2 alterations were concentrated in a subset of genes including *KRAS*, *ATM*, *EGFR*, and *PIK3CA*, but were also widely distributed across 36 other genes (Fig. 4b). At the patient-level, cases with Tier 1 and 2 alterations accounted for 37.7%, 41.8%, 51.1%, and 19.4% of patients that had emergent mutations with PDAC, CCA, EGC, and HCC, respectively (Fig. 4c). We also examined whether emergent alterations would enhance trial eligibility by determining if mutations could be matched to active clinical trials in clinicaltrials.gov at the time of progression and with an enrolling site within the same state as the patient. This analysis demonstrated that emergent alterations increased trial eligibility in 27%, 20%, 20%, and 14% of all patients with PDAC, CCA, EGC, and HCC, respectively (Fig. 4d).

### Outcomes after receiving matched therapies to emergent alterations

To assess clinical outcomes among patients who received therapies matched to emergent alterations, we retrospectively reviewed a separate cohort of 379 patients with advance non-colorectal gastrointestinal cancers who had liquid biopsies performed as routine care at two institutions. Serial liquid biopsies including at the time of treatment progression was performed on 61 of these patients, of which 20 had emergent alterations that were therapeutically relevant. Chart review revealed that 5 patients had a change in treatment as a result of an emergent ctDNA alteration (Supplementary Table 1).

**Fig. 5:**
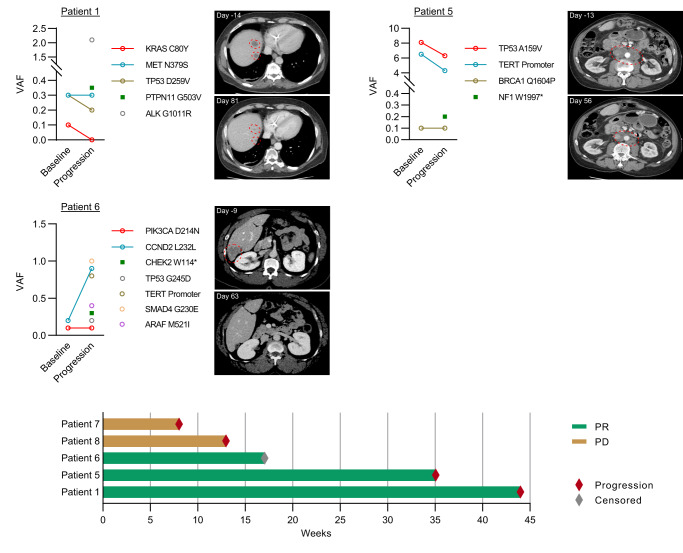
Outcomes of patients receiving matched therapies to emergent ctDNA alterations. ctDNA alterations at baseline and progression are shown in dot plots with emergent alterations targeted by matched therapies shown as green squares. Representative target lesions denoted by red dotted outlines are shown on axial slices from computerized tomography scans prior to and after receiving matched therapies. D1 denotes the first day of treatment. Column chart summarizes tumor responses and progression-free survival of five patients in the case series who received matched therapies. PR partial response, PD progressive disease.

Patient 1 had metastatic CCA with an emergent PTPN11 G503V alteration that was subsequently treated with nivolumab, an anti-PD-1 antibody. Although immune checkpoint inhibitors are associated with low response rates in CCAs, mutations in *PTPN11* in glioblastoma, an immunotherapy refractory cancer, is associated with clinical benefit after treatment with anti-PD-1 antibodies^35^. Treatment led to a partial response lasting nearly 11 months (Fig. 5). To assess the generalizability of *PTPN11* alterations as a predictive marker of immunotherapy benefit in primary liver cancers, we identified additional three patients with primary liver cancers and pathogenic *PTPN11* mutations detected at the time of diagnosis who were treated with immune checkpoint inhibitors as any line of therapy including two patients with CCA (Patient 2 and 4), and one patient with HCC (Patient 3). Clinical benefit with anti-PD-1 antibodies was observed in Patient 2 and 3 who had stable disease and a partial response, respectively, while Patient 4 had progressive disease at first tumor assessment (Supplementary Table 1).

Patient 5 had metastatic HCC with an emergent pathogenic *NF1* mutation for which the patient was initiated on regorafenib (Fig. 5). In contrast to other tyrosine kinase inhibitors used in HCC, regorafenib is a potent inhibitor of RAF kinases which are hyperactivated as a result of *NF1* inactivation^36^. The patient achieved a partial response with >50% reduction in tumors which continues to persist after 8 months.

Patient 6 had metastatic HCC with an emergent pathogenic *CHEK2* mutation for which the patient was initiated on olaparib as prior studies have shown that cancers, particularly of the prostate, with DNA repair defects may be sensitive to poly adenosine diphosphate-ribose polymerase inhibitors^37^. The patient’s best overall response was a partial response with a complete response in some lesions (Fig. 5).

Patients 7 and 8 had CCA and PDAC, respectively, who had emergent hotspot *PIK3CA* mutations for which alpelisib, a PIK3CA inhibitor, was initiated (Supplementary Table 1). Both patients had progressive disease at their first tumor re-assessment.

In summary, three of five patients with actionable emergent ctDNA alterations who received matched therapies experienced disease control (three partial responses). No patients were able to be enrolled in a prospective clinical trial and no significant toxicities leading to treatment discontinuation were observed with matched therapies. Clinical benefit from matched therapy lasting at least 6 months was observed in two patients.

## Discussion

The genetics underlying treatment-resistant non-colorectal gastrointestinal cancers have not been previously well-elucidated. Tumor-naive liquid biopsies may faithfully capture unanticipated changes in the landscape and distribution of genetic alterations resulting from the selective pressures of systemic therapies^5^. Our study leverages this feature to provide the largest systematic analysis of serial liquid biopsies to date to illustrate how cancer genomes evolve in response to treatment in multiple gastrointestinal histologies.

Because ctDNA provides real-time detection of clonal changes, serial liquid biopsies are more informative than baseline molecular snapshots in elucidating predictive rather than prognostic markers of treatment in a patient population. Across our cohort, *TP53* alterations were associated with resistance in all cancer types which is consistent with *TP53* mutations as mechanisms of resistance to chemotherapy, the mainstay of treatment for PDAC, CCA, and EGCs^38^. However, multiple other resistance alterations were also uncovered in this study including *CCNE1* alterations in EGC, *GNAS* alterations in HCC, and *CTNNB1* mutations in CCA. We also identified *PIK3CA* alterations as potential resistance markers in PDAC, and a similar association has been reported in breast cancers treated with chemotherapy^39^.

Emergent ctDNA alterations were highly prevalent and associated with therapeutic actionability in a substantial proportion of patients in our cohort based on a holistic molecular tumor board review. Whether these determinations may translate into improved patient outcomes is unknown, though patients receiving therapies based on molecular tumor board recommendations have been associated with tumor responses and enhanced survival in real-world studies^40,41^. We also showed that between 14% to 27% of patients across histologies in our cohort were eligible for additional trials in the same state of residence. This provides a practical and objective measure of the benefit of serial ctDNA testing. Thus, serial liquid biopsies may be clinically meaningful in a substantial subset of patients with gastrointestinal cancers, and their value could increase with the growth of biomarker-directed therapies. The identification of emergent fusions is remarkable as these alterations are crucial driver events associated with high responses to targeted therapies^42^. However, the emergent fusions we detected remain to be validated as confirmatory methods including RNA sequencing or fluorescence in situ hybridization were not performed. Notably, emergent alterations arose in a meaningful proportion of patients who had declining VAFs in baseline alterations, which demonstrates an advantage of ctDNA testing that is not informed by only known alterations previously detected. There was no difference in time to progression between cases with emergent and non-emergent alterations, suggesting high variance in the development of emergent mutations. This may be explained by divergent proliferation rates among clonal populations harboring resistant mutations across cancers which indicates that time to progression may not readily predict emergent alterations.

Emergent ctDNA alterations likely originate from subclonal populations that survive selection mediated by treatment and then undergo sufficient expansions until ctDNA can be detected. Thus, the classification of emergent ctDNA alterations is likely tied to the sensitivity of a liquid biopsy assay. However, the clinical ctDNA assay utilized in this study has high sensitivity (down to 0.01% VAF) which is largely constrained by specimen and technological limitations shared by other next-generation sequencing liquid biopsy platforms. Whether emerging methods with increased sensitivity such as enrichment strategies may increase or decrease emergent ctDNA alteration detection remains to be clarified^43^. Nevertheless, the abundance of pathogenic emergent ctDNA alterations using a validated liquid biopsy assay suggests that prior genomic profiles derived from single timepoints are inadequate portrayals of molecular alterations and clonal structures in cancers.

The degree in which TMB may fluctuate as a result of treatment has not been well studied with a few exceptions including temozolomide induced hypermutation^44^. Our results indicate that TMB often changes in response to treatment, but there was no clear relation to clinical or molecular factors examined. Nonetheless, our results show that a subset of patients who would have been previously ineligible for immune checkpoint inhibitors based on the current FDA approved TMB cutoff may subsequently be eligible based on serial ctDNA monitoring. It remains to be investigated whether changes in TMB may reflect sensitivity to agents that are more effective in TMB-high cancers including immune checkpoint inhibitors^45^.

The clinical benefit observed in a subset of patients receiving matched therapies to emergent ctDNA alterations suggests that some emergent ctDNA alterations may have therapeutic relevance. Notably, our results suggest that *PTPN11* loss-of-function mutations may be a cancer-agnostic predictive marker of immunotherapy benefit. In addition, olaparib has not been previously reported to be clinically effective in HCC, but our case study indicates that DNA repair defects may be predictive of sensitivity to poly ADP-ribose polymerase inhibitors in HCC despite their low frequency in this cancer type. Uptake of matched therapies based on ctDNA results was low and did not lead to trial enrollment in the two institutions analyzed, which may be due to provider ambivalence, lack of biomarker-directed trials, and limited insurance coverage for off-label therapies. Our case series showed no association between the benefit of matched therapies and the VAF of the targeted alteration, but further studies are needed to identify clinical and molecular factors that affect the benefit of targeting emergent ctDNA alterations.

This study was retrospectively designed, focused on select cancer-associated genes, and concurrent tissue analyses from multi-site biopsies in our patients were not possible given the nature of our real-world dataset. There was also limited clinical annotation in the national cohort including an absence of precise treatment dates, dates of progression, and therapies used. However, during the period of the study, the standard of care treatments for PDAC, CCA, and EGC was largely chemotherapy, and immunotherapies or targeted therapies in HCC. Future studies within treatment-defined patient cohorts are needed to determine if the findings presented are generalizable. Additionally, the contribution of alterations from clonal hematopoiesis in this study cannot be excluded, although frequently mutated genes in clonal hematopoiesis including *DNMT3A*, *TET2*, *PPM1D*, *ASXL1, GNB1*, *CBL*, *SRSF2*, and *SF3B1* which account for over 90% of mutations associated with clonal hematopoiesis were not assessed in the ctDNA assay used^46,47^. Our study also focused on a second serial liquid biopsy, and the utility of ctDNA testing at additional timepoints needs to be explored. Notwithstanding these limitations, this study provides a comprehensive landscape of genetic alterations and their dynamics among refractory non-colorectal gastrointestinal cancers in a large cohort of patients. These results represent an important resource for understanding evolutionary changes that underlie treatment resistance and demonstrate the high prevalence and potential therapeutic relevance of subclonal resistance alterations.

## Methods

### Study population

After receiving institutional review board (IRB) approval at the University of Texas Southwestern Medical Center (UTSW), a retrospective analysis of deidentified reports was performed on a national cohort of 613 eligible patients who received ctDNA testing (Guardant360, Guardant Health) performed as part of routine clinical care. Eligibility criteria included a diagnosis of PDAC, CCA, EGC, or HCC, two ctDNA tests that were of the same versions between 1 October 2020 to 1 October 2021, and at least one detectable alteration on either the baseline or progression liquid biopsy. A subset of patients in this cohort (*N* = 35) had clinical and pathologic data accessible. Tissue specimens for these patients were identified only if they had been collected within 3 months of the baseline ctDNA collection and TMB analysis was determined from either targeted (FoundationOne) or exome sequencing (Caris Life Sciences). Tumor volumes were determined from radiographic images including computed tomography and magnetic resonance imaging performed within 4 weeks of liquid biopsies. To calculate tumor volumes, three-dimensional measurements (diameters on the axial, sagittal, and coronal plane) of all lesions for every lesion were used to calculate the volume of an ellipsoid (4/3 × π × width × length × height) which has been shown to approximate tumor volumes^48,49^.

A separate cohort analysis was performed on patients who received cancer care between 1 August 2019 and 1 May 2022 for advanced PDAC, CCA, EGC, or HCC at UTSW and Parkland Hospital and had liquid biopsies analyzed using commercial assays including Guardant, Tempus, or Foundation as part of routine clinical care. Patients with at least two ctDNA tests that were of the same versions were identified and clinical data including demographics, treatment history, tumor responses, and survival outcomes were abstracted from the electronic medical record. This analysis was performed in accordance with Good Clinical Practices and the Declaration of Helsinki and approved by the UTSW IRB.

### Sample collection and ctDNA sequencing

Liquid biopsies were collected in two 10 mL tubes of whole blood per individual in Streck Cell-Free DNA Blood Collection (Streck) tubes. Samples were shipped to a Clinical Laboratory Improvement Act (CLIA)-certified, College of American Pathologists-accredited laboratory (Guardant Health, Redwood City, CA). After double ultracentrifugation, a minimum of 5 ng of cell-free DNA was isolated for library preparation. As previously described, the Guardant360 assay is a targeted high throughput hybridization-based capture technology for the detection of single nucleotide variants, insertions, and deletions in 73 or 84 genes by paired-end synthesis-sequencing using the NextSeq 500 and/or HiSeq 2500 platforms (Illumina, Inc.)^50^. Only tests that had ctDNA analyzed using the same version of the assay were included, and only the two most recent tests in the study period were analyzed.

### ctDNA analysis

Putative germline mutations including variants identified by allele fractions between 40% and 60%, prior annotation as germline mutations, and manual review were excluded from our analyses. Putative clonal hematopoiesis of indeterminate potential mutations, identified as mutations commonly annotated in the literature and in sequencing of healthy normal donors, were also excluded^32,50^. In addition, the most frequent genes associated with clonal hematopoiesis including *DNMT3A*, *TET2*, *PPM1D*, *ASXL1, GNB1*, *CBL*, *SRSF2*, and *SF3B1* are not represented in the Guardant360 gene panel. Single nucleotide variants were considered pathogenic based on a consensus of calls from prediction algorithms in VarSome. TMB was calculated by counting all somatic nonsynonymous and single nucleotide variants and delins across 1 Mb of coding regions and then algorithmically adjusted to correct for confounding biological and technical sample features as previously described^50^. Microsatellite instability was determined in the Guardant360 assay by sequencing of 90 microsatellite loci as previously described^51^. To examine changes in ctDNA over time, alterations were classified as emergent (not detectable at baseline but detectable at any VAF at progression, increasing (detectable at baseline with rise in VAF by 20% or greater at progression), stable (detectable at baseline with less than 20% increase or decrease in VAF at progression), decreasing (detectable at baseline with decline in VAF by 20% or greater at progression), or lost (detectable at baseline at any VAF but not detectable at progression). CNAs were first categorized on a semi-quantitative scale as low (below the 50th percentile of amplifications detected by the assay), medium (between the 50th and 90th percentile) or high (above the 90th percentile). CNAs were then classified as emergent (not detectable at baseline but detectable at any amplification at progression), increasing (detectable at baseline with an increase in amplification category), stable (detectable at baseline with no change in amplification category), decreasing (detectable at baseline with a decrease in amplification category), or lost (detectable at baseline but not detectable at progression).

### Molecular tumor board

The UTSW molecular tumor board is directed by a medical oncologist experienced in clinical trials, genomics, and immunotherapy who moderates a biweekly conference. Other attendees include medical oncologists, pathologists, geneticists, pharmacists, and research staff. To determine the therapeutic relevance of emergent ctDNA alterations, the molecular tumor board was convened ad-hoc to determine tumor-specific tiers of evidence to support actionability. Recommendations were based on discussions regarding the clinical relevance of the alteration, and the likelihood of treatment benefit from targeted therapies based on literature reviews for preclinical or clinical evidence. Determinations were also informed by the use of variant annotation databases such as OncoKB, clinicaltrials.gov, and Varsome.

### Statistical methods

Clinical and molecular characteristics were summarized by descriptive statistics. The proportion of patients with resistant and sensitive alterations, and patients with increasing/stable and decreasing VAF in baseline alterations were compared using the Fisher exact test. Pearson correlation coefficient was calculated to determine the linear relationship between VAF and weeks until progression, and TMB at baseline and progression. Comparisons of means were performed using the Student’s *t* test or the Mann–Whitney test for data without a Guassian distribution. Tumor response was determined using RECIST 1.1 criteria. Statistical analyses were performed using GraphPad Prism version 9.3.1 (GraphPad) and SPSS version 24 (IBM).

### Reporting summary

Further information on research design is available in the Nature Portfolio Reporting Summary linked to this article.

### Supplementary information

Supplementary Information

Reporting Summary

### Source data

Source Data

## Data Availability

Restrictions apply to the availability of ctDNA sequences in the national cohort which was obtained from Guardant Health due to data privacy regulations and restrictions for use in the patient consent form. Requests are to be made to David Hsiehchen (gbtwnow@gmail.com) describing the nature of the proposed research and the extent of data requirements. Data recipients may require a collaborative research agreement, which describes the conditions for data release and requirements for data transfer, storage, archiving, publication, and intellectual property. Source data are provided in this paper.
