# Author Correction: Genetic features and therapeutic relevance of emergent circulating tumor DNA alterations in refractory non-colorectal gastrointestinal cancers

**DOI:** 10.1038/s41467-023-43052-1

**Published:** 2023-11-22

**Authors:** David Hsiehchen, Leslie Bucheit, Dong Yang, Muhammad Shaalan Beg, Mir Lim, Sunyoung S. Lee, Pashtoon Murtaza Kasi, Ahmed O. Kaseb, Hao Zhu

**Affiliations:** 1 Division of Hematology and Oncology, Department of Internal Medicine, University of Texas Southwestern Medical Center, Dallas, TX USA; 2 Guardant Health Inc, Redwood City, CA USA; 3 Department of Gastrointestinal Medical Oncology, University of Texas MD Anderson Cancer Center, Houston, TX USA; 4 Weill Cornell Medicine, Englander Institute of Precision Medicine, Meyer Cancer Center, New York, NY USA

**Keywords:** Bile duct cancer, Cancer therapeutic resistance, Pancreatic cancer, Hepatocellular carcinoma, Cancer genomics

Correction to: *Nature Communications*; 10.1038/s41467-022-35144-1; Article published online 03 December 2022

The original version of this Article contained an error in the Supplementary Information file, in which Supplementary Figures 15 and 16 were mistakenly included. These figures were replaced by Supplementary Table 1 during revision. The HTML has been updated to include a corrected version of the Supplementary Information. The in-text citations have also been updated accordingly, and this has been corrected in both the PDF and HTML versions of the Article.

The original version of this Article contained an error in the Methods section, which incorrectly read ‘A separate cohort analysis was performed on patients who received cancer care between 1 August 2019 and 1 May 2022 for advanced PDAC, CCA, EGC, or HCC at UTSW and Parkland Hospital and had liquid biopsies analyzed using the Guardant360 assay as part of routine clinical care.’ The correct version replaces this sentence with ‘A separate cohort analysis was performed on patients who received cancer care between 1 August 2019 and 1 May 2022 for advanced PDAC, CCA, EGC, or HCC at UTSW and Parkland Hospital and had liquid biopsies analyzed using commercial assays including Guardant, Tempus, or Foundation as part of routine clinical care’. This has been corrected in both the PDF and HTML versions of the Article.

The original version of this Article contained an error in Fig. 5, in which Patient 9 was included as an example. The baseline testing used on this patient used a modified ctDNA test, which did not directly correlate to the post-treatment testing. The authors have therefore chosen to remove this patient from the manuscript. The text of the Results section and the legend of Figure 5 have also been edited to remove the description of this patient as follows ‘Patient 9 had CCA with an emergent inactivating TSC1 mutation for which the patient was initiated on everolimus, an inhibitor of the mammalian target of the rapamycin signaling pathway. The patient experienced stable disease for nearly 5 months (Fig. 5)’. The following sentence was also corrected to update these results, ‘Chart review revealed that 6 patients had a change in treatment as a result of an emergent ctDNA alteration (Supplementary Table 1). The correct version replaces ‘6 patients’ with ‘5 patients'. Similarly, ‘In summary, five of six patients with actionable emergent ctDNA alterations who received matched therapies experienced disease control (three partial responses and one stable disease)’ was corrected to ‘In summary, four of five patients with actionable emergent ctDNA alterations who received matched therapies experienced disease control (three partial responses)'. The new legend of Figure 5 now reads “Column chart summarizes tumor responses and progression-free survival of five patients in the case series who received matched therapies. PR partial response, PD progressive disease”. Patient 9 data has also been removed from the updated Source Data file and Supplementary Table 1. This has been corrected in both the PDF and HTML versions of the Article.

The original version of this Article contained an error in Fig. 5, in which the MRI images of Patient 5 indicated stable disease. These images have been replaced to illustrate the shrinking of lymph node metastasis within the patient. This has been corrected in both the PDF and HTML versions of the Article.

### Supplementary information

Supplementary Information

### Source data

Source Data

