# Supplementary material for: Genetic features and therapeutic relevance of emergent circulating tumor DNA alterations in refractory non-colorectal gastrointestinal cancers
Source: Nat Commun. 2022 Dec 3;13:7477. doi: 10.1038/s41467-022-35144-1 (PMC9719461; doi:10.1038/s41467-022-35144-1)
Supplement: Supplementary file 1 — Supplementary Information [file 41467_2022_35144_MOESM1_ESM.pdf]

Supplementary Table 1. Characteristics and history of patients receiving matched therapies to emergent alterations.

Case	Clinical history prior to detection of emergent alteration	Alteration and matched therapy
1	Metastatic CCA treated with gemcitabine plus cisplatin for 3 months with progression.	*PTPN11*; nivolumab
5	Metastatic HCC treated with pembrolizumab and lenvatinib.	*NF1*; regorafenib
6	Metastatic HCC treated with nivolumab and lenvatinib.	*CHEK2*; olaparib
7	Advanced CCA treated with gemcitabine plus cisplatin, nivolumab, and FOLFOX	*PIK3CA*; alpelisib
8	Metastatic PDAC treated with mFOLFIRINOX and gemcitabine plus nab-paclitaxel	*PIK3CA*; alpelisib

Supplementary Figure 1. Association between changes in ctDNA VAF, tumor markers, and tumor volumes.

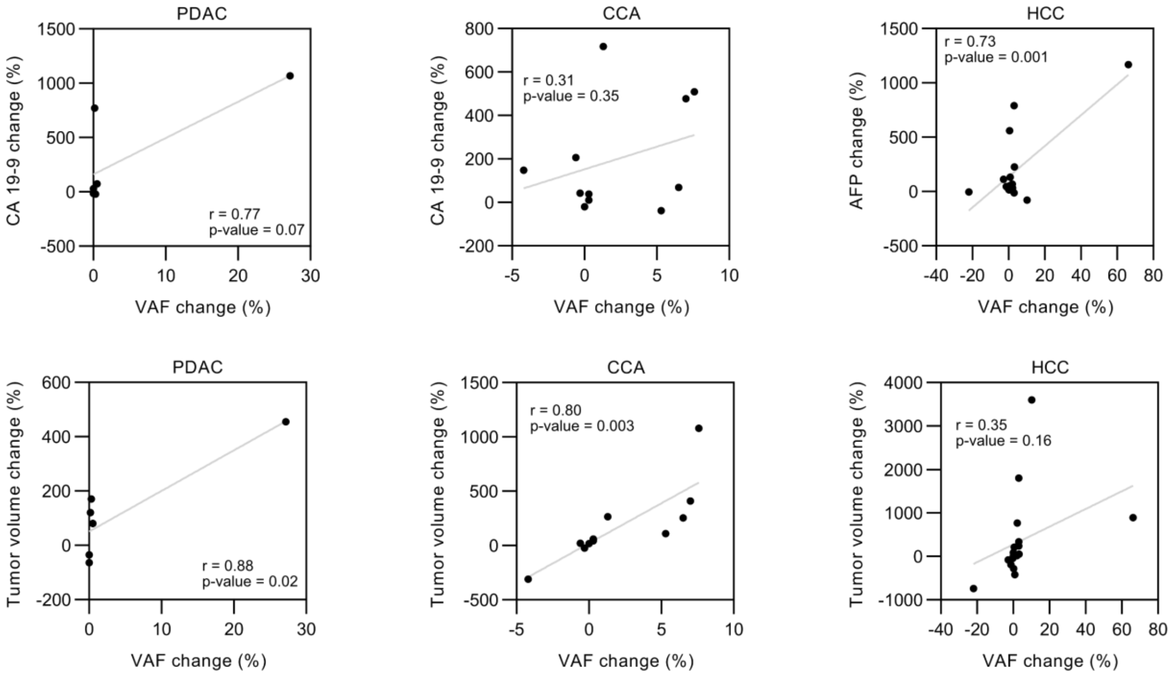

Percent change in ctDNA maximal VAF were plotted against percent change in serum tumor markers and tumor volumes. Correlations were assessed using Pearson's r and 2-tailed p-values.

Supplementary Figure 2. Types of ctDNA alterations at baseline and progression.

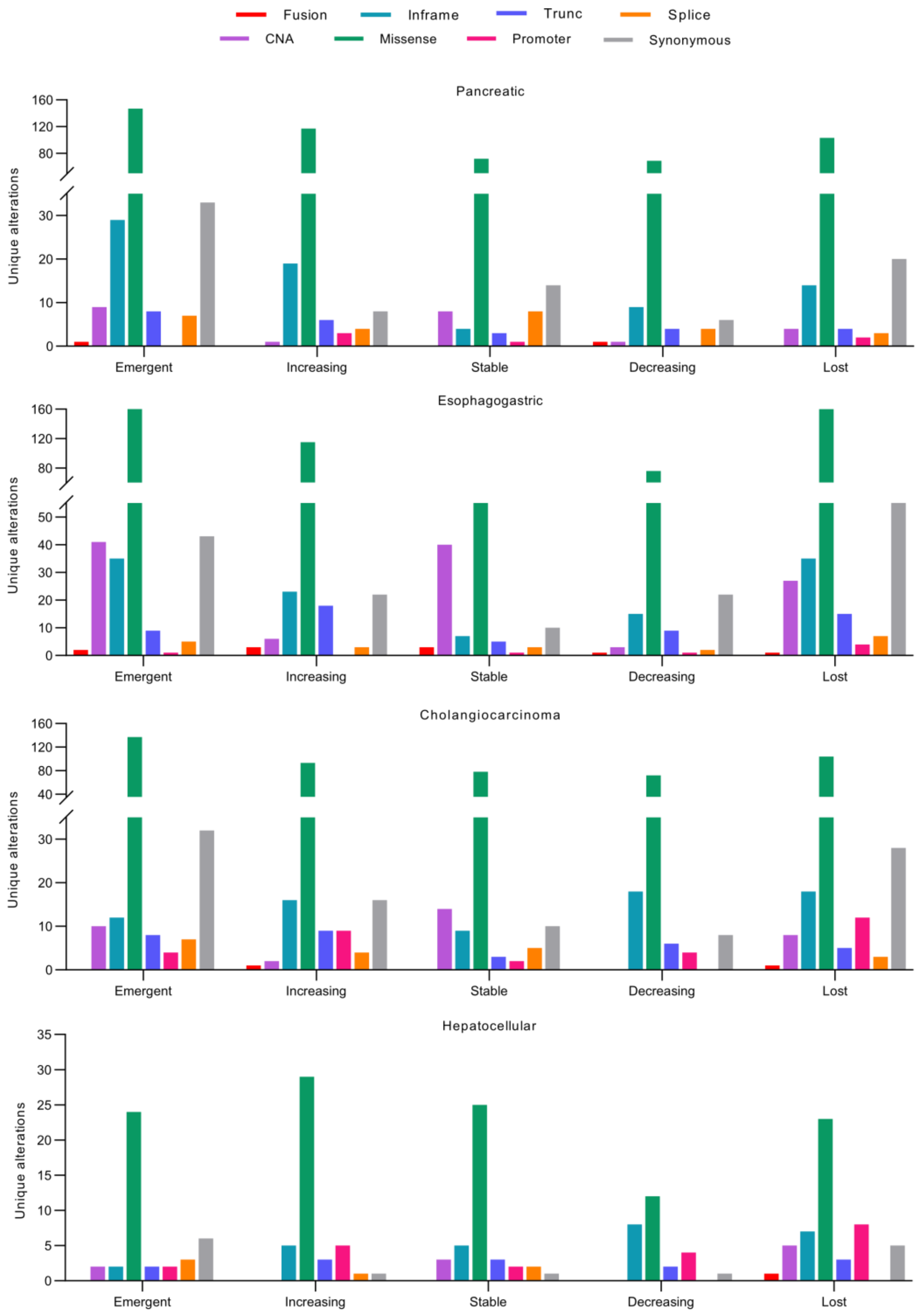

Distribution of ctDNA alterations including synonymous mutations by type in each histology.

Supplementary Figure 3. Genes associated with emergent ctDNA alterations.

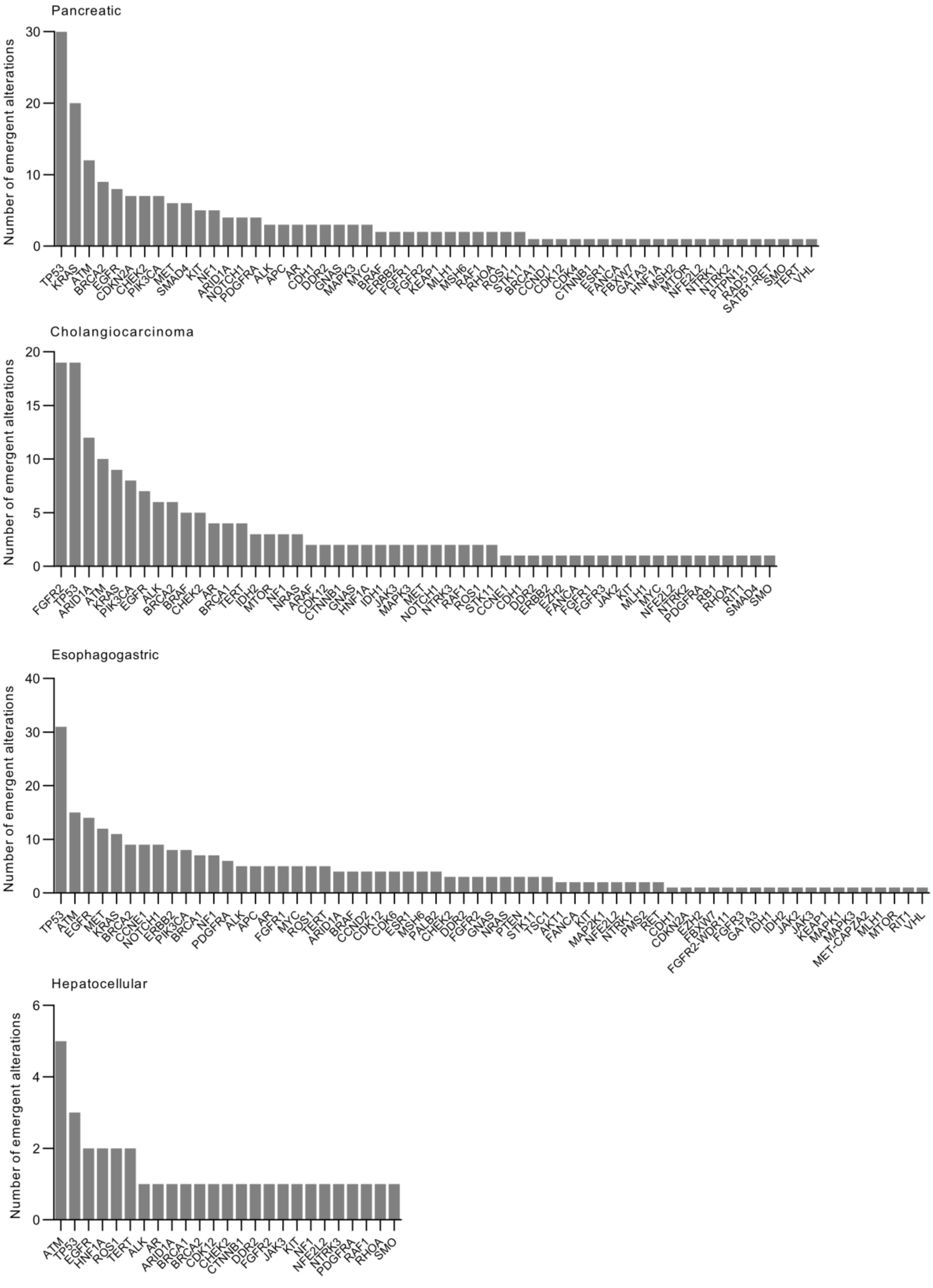

Distribution of emergent ctDNA alterations among genes in each histology.

Supplementary Figure 4. VAF of emergent KRAS mutations.

Patient	Baseline driver gene VAF		Emergent KRAS alteration VAF	
P-16	0.30	TP53	0.10	KRAS Q61R
P-29	0.10	TP53	1.60	KRAS G12V
P-31	0.60	ATM	0.10	KRAS G12V
P-34	0.20	EGFR	0.30	KRAS G12D
P-43	0.10	RET	0.06	KRAS G12D
P-49	0.10	NF1	0.03	KRAS G12V
P-59	0.20	KRAS V14I	0.03	KRAS G12D
P-60	0.40	BRCA1	0.50	KRAS G12S
P-64	0.80	CHEK2	0.50	KRAS G12D
P-69	0.50	CHEK2	0.09	KRAS G12D
P-72	47.80	BRAF	0.10	KRAS G12D
P-76	0.90	RAF1	0.04	KRAS G12R
P-85	0.50	ARAF	0.02	KRAS G12D

0.00 1.00

Maximal VAF of baseline ctDNA alterations are shown among all patients with PDAC and emergent KRAS mutations.

Supplementary Figure 5. Emergence of polyclonal FGFR2 mutations.

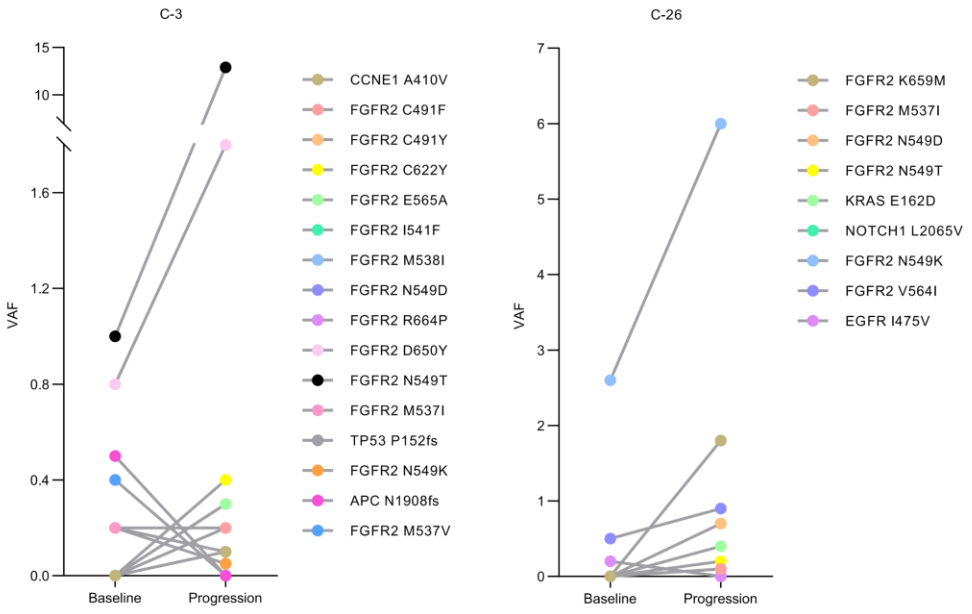

Two patients with BTC had emergent polyclonal FGFR2 ctDNA mutations consistent with acquired resistance to FGFR2 inhibitors.

Supplementary Figure 6. Relative clone size of emergent ctDNA alterations.

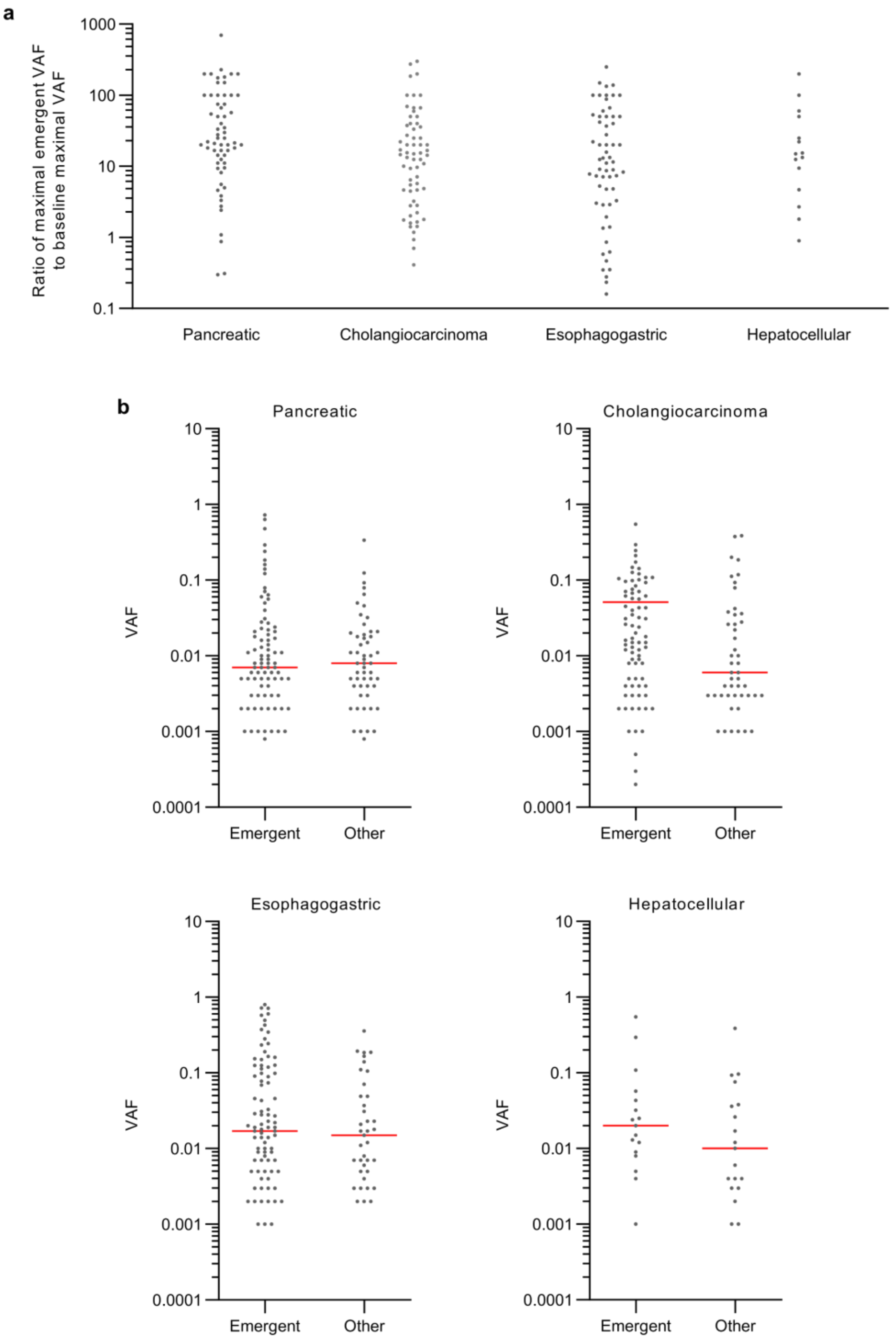

a Dot plot shows the ratio of the maximal VAF of any emergent ctDNA alteration to the maximal VAF of any baseline ctDNA alteration in each histology. Each dot represents a patient with emergent ctDNA alterations.

b Maximal VAF at baseline was compared between cases with and without emergent alterations using the two-sided Mann-Whitney test in PDAC (p-value=0.55), CCA (p-value=0.04), EGC (p-value=0.77), and HCC (p-value=0.23).

Supplementary Figure 7. Time to progression in cases with emergent or non-emergent ctDNA alterations.

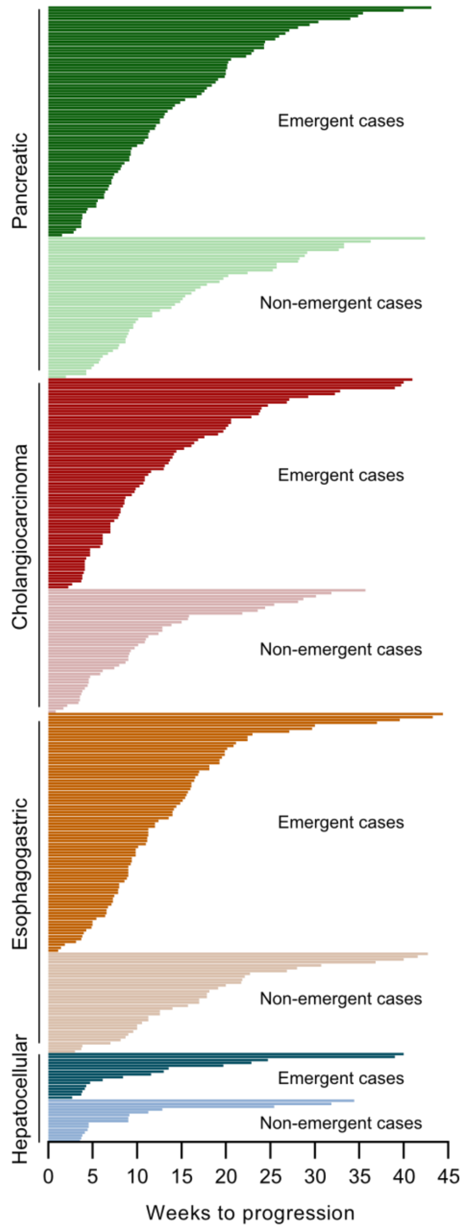

Weeks until progression among patients with emergent or non-emergent ctDNA alterations are shown in a bar chart. A two-sided t-test was used to assess for differences in time to progression in emergent and non-emergent cases for each cancer type. No differences in time to progression was noted within cancer types between emergent and non-emergent cases using the t-test with the exception of EGC where emergent cases had shorter time to progression (13.7 weeks versus 17.8weeks, t-test p-value=0.03).

Supplementary Figure 8. ctDNA changes and time to progression.

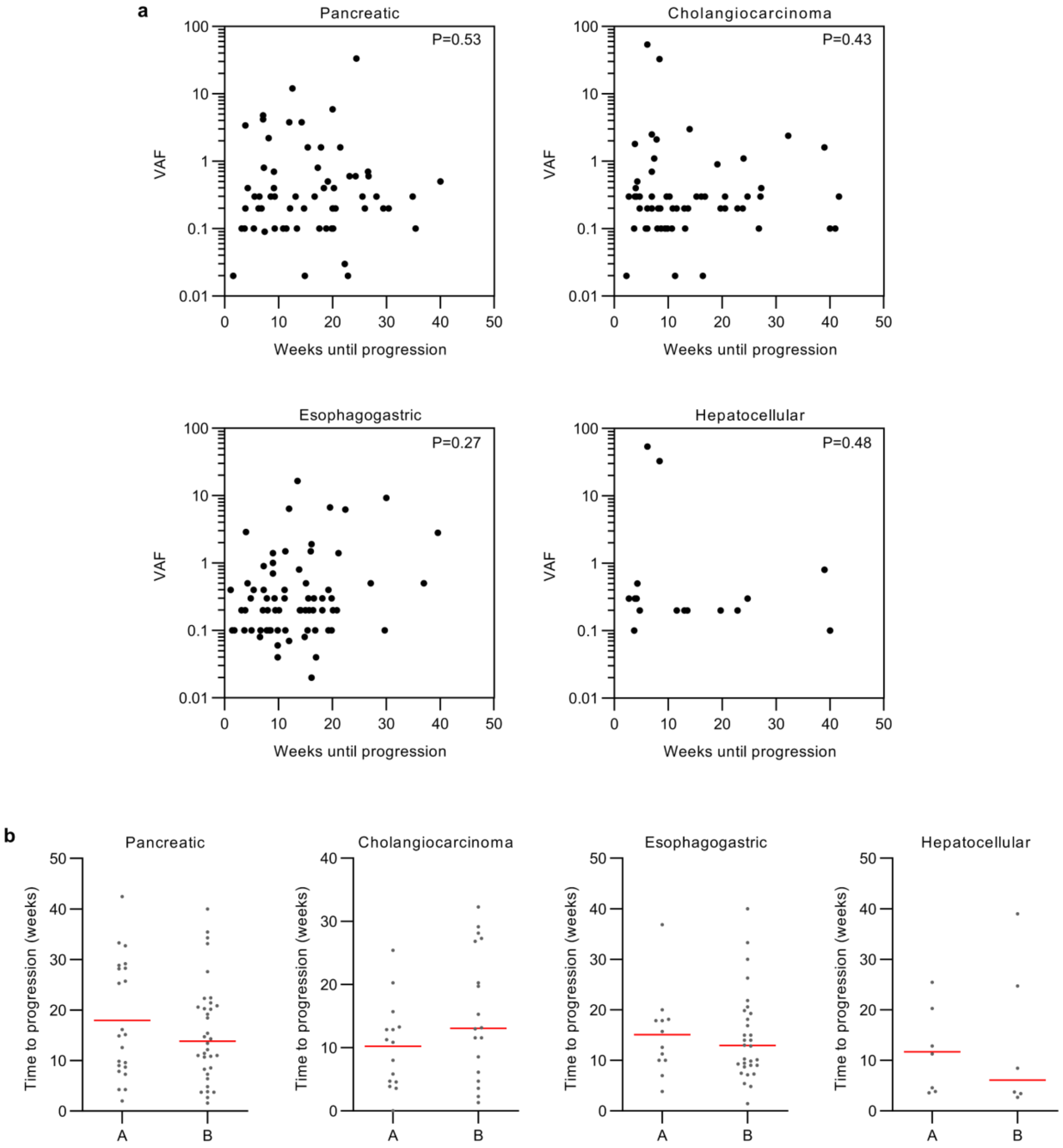

a Pearson correlation coefficient was calculated to assess the relationship between the maximal VAF of any emergent ctDNA alteration and time to progression. Time to progression data was available in a subset of patients. Correlations were assessed using Pearson's r and 2-tailed p-values.

b Time to progression was compared between patients with rising maximal VAFs of baseline alterations but without emergent alterations, and patients with emergent alterations plus declining maximal VAFs of baseline alterations using the two-sided Mann-Whitney test in PDAC (p-value=0.51), CCA (p-value=0.15), EGC (p-value=0.74), and HCC (p-value=0.63).

Supplementary Figure 9. Emergent fusion/rearrangements detected.

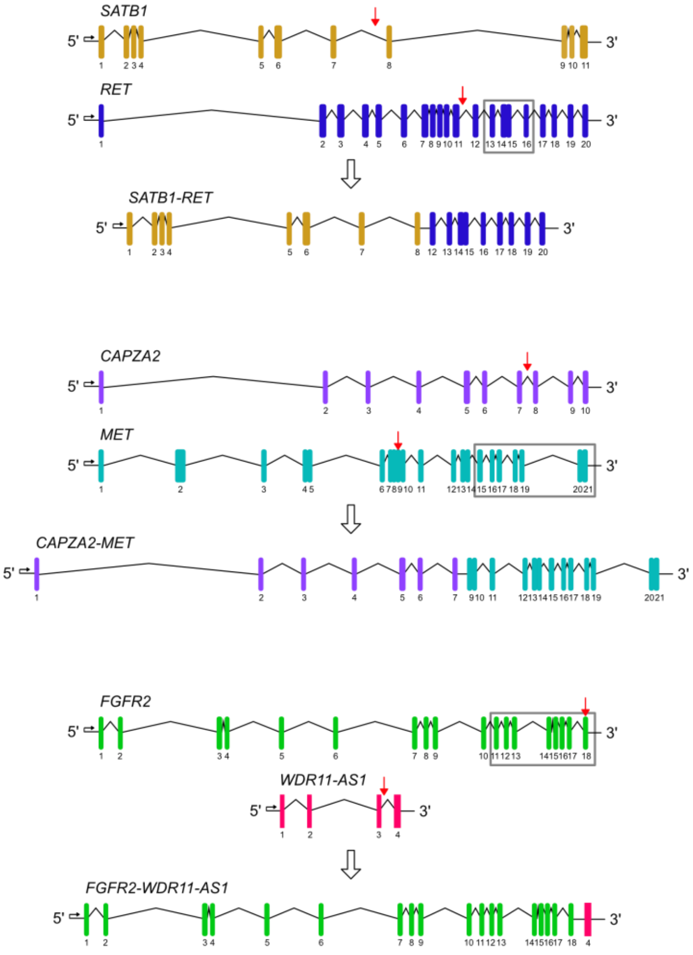

Schematic diagrams of the predicted *SATB1-RET*, *CAPZA2-MET*, and *FGFR2-WDR11-AS1* fusions and breakpoints. Exons encoding the RET, MET, and FGFR2 kinase domains are highlighted in the grey box.

Supplementary Figure 10. Comparisons of baseline tissue, baseline ctDNA, and emergent ctDNA alterations in a patient subset.

Cancer type	Exclusive baseline tissue alterations (A)	Exclusive baseline ctDNA alterations (B)	Exclusive emergent ctDNA alterations (C)	Overlap between A and B	Overlap between A and C
PDAC	0	3	1	4	0
PDAC	0	2	1	1	0
PDAC	1	1	1	4	0
HCC	0	2	3	5	0
HCC	0	0	2	5	0
HCC	0	0	1	3	0
HCC	1	1	0	2	1
HCC	0	1	2	2	0
HCC	0	0	0	1	0
CCA	0	2	0	3	0
CCA	2	0	1	1	0
CCA	0	2	2	3	0
CCA	0	0	1	3	0
CCA	0	0	0	2	0
CCA	0	0	1	4	0
CCA	0	2	1	4	1

Heat map shows the number of alterations that are exclusive to baseline tissue, baseline ctDNA, or emergent ctDNA, as well as the number of overlapping alterations between baseline tissue and baseline ctDNA, and baseline tissue and emergent ctDNA.

Supplementary Figure 11. TMB at baseline and progression are not significantly different.

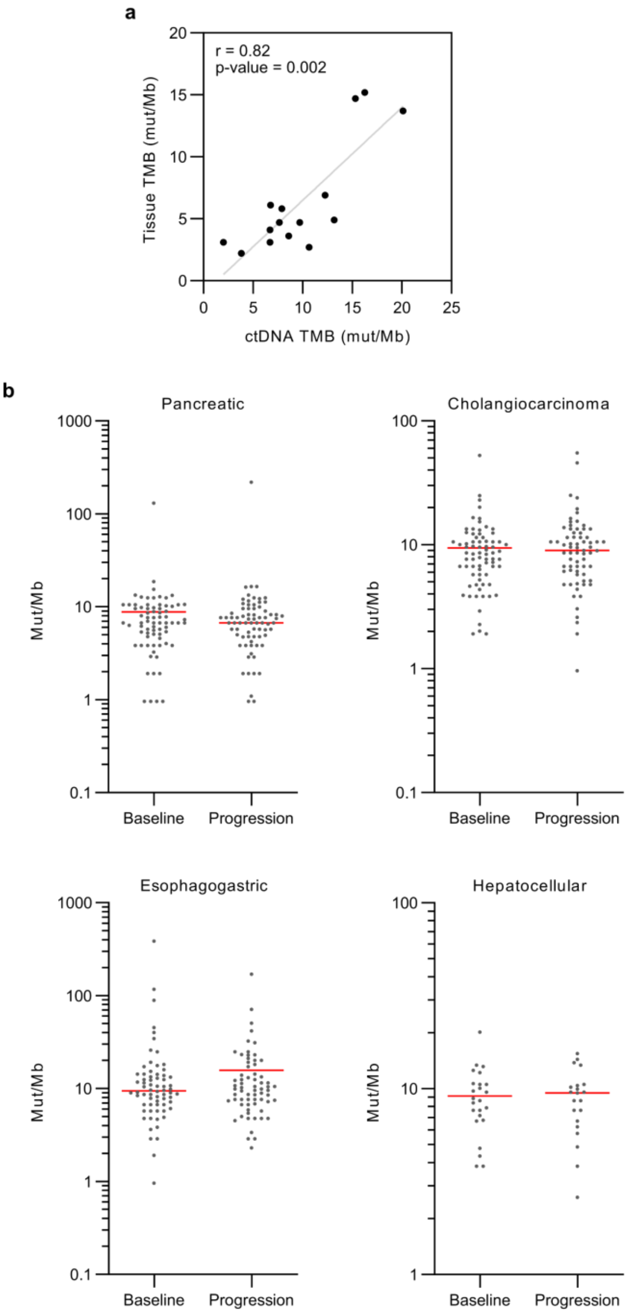

a Scatter plot shows the relationship between TMB determined from baseline tissue versus baseline ctDNA. A correlation was assessed using Pearson's r and a 2-tailed p-value.
b Dot plot shows the distribution of TMB at baseline and progression in each histology. Red lines show the mean TMB. Two-sided Student's t-test showed no significant difference between the mean TMB at baseline and progression for any histology.

Supplementary Figure 12. Correlations between TMB at baseline and progression.

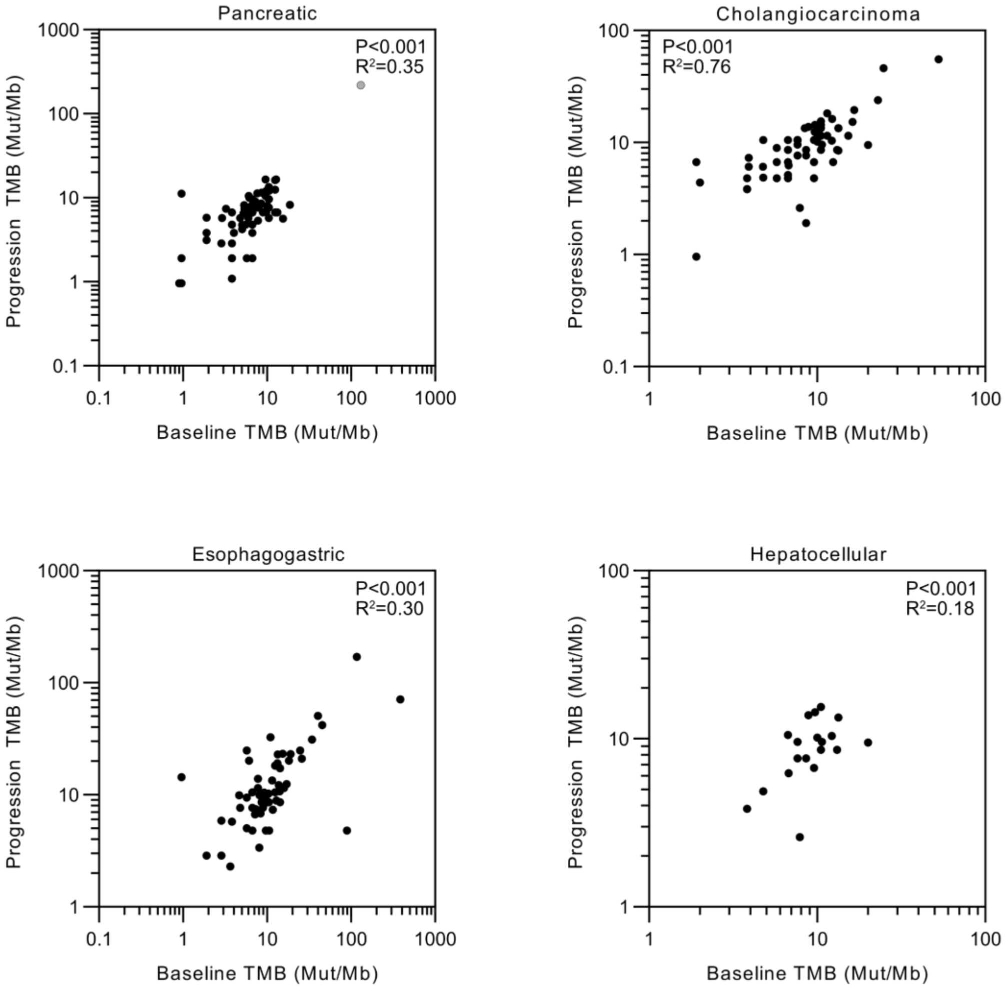

Pearson correlation coefficient was calculated to assess the relationship between TMB at baseline and progression. R^2 denotes the coefficient of determination which is the proportion of the variance in the dependent variable explained by the independent variable. Correlations were assessed using Pearson's r and 2-tailed p-values.

Supplementary Figure 13. Impact of TMB changes among TMB-high cancers.

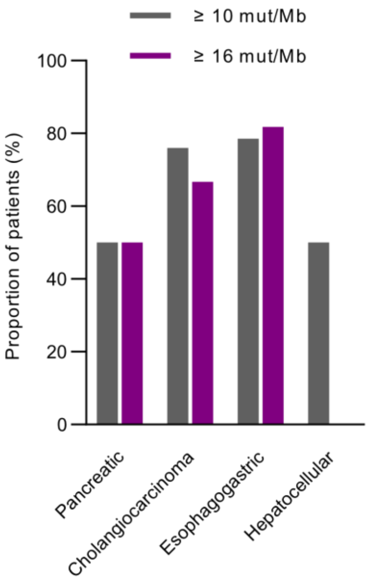

Column charts indicate the proportion of patients with a baseline TMB of at least 10 or 16 mut/Mb who had a TMB at progression of at least 10 or 16 mut/Mb.

Supplementary Figure 14. Therapeutic relevance of ctDNA alterations.

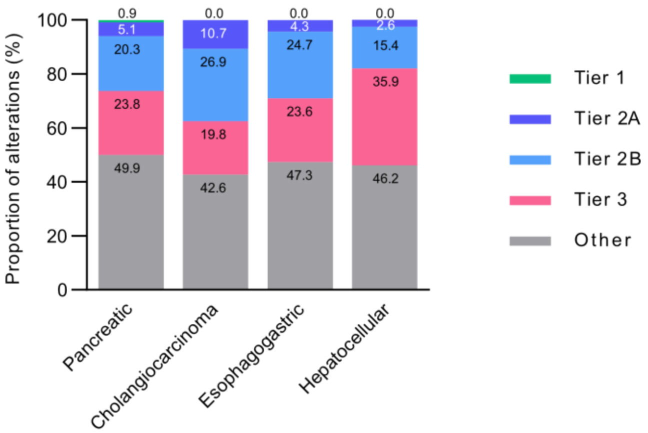

Distribution of emergent ctDNA alterations by tier of evidence supporting therapeutic relevance.
