# Supplementary material for: Genetic features and therapeutic relevance of emergent circulating tumor DNA alterations in refractory non-colorectal gastrointestinal cancers
Source: Nat Commun. 2022 Dec 3;13:7477. doi: 10.1038/s41467-022-35144-1 (PMC9719461; doi:10.1038/s41467-022-35144-1)
Supplement: Supplementary file 2 — Reporting Summary [file 41467_2022_35144_MOESM2_ESM.pdf]

nature portfolio

Corresponding author(s): David Hsiehchen

Last updated by author(s): Oct 25, 2022

Reporting Summary

Nature Portfolio wishes to improve the reproducibility of the work that we publish. This form provides structure for consistency and transparency in reporting. For further information on Nature Portfolio policies, see our Editorial Policies and the Editorial Policy Checklist.

Statistics

For all statistical analyses, confirm that the following items are present in the figure legend, table legend, main text, or Methods section.

n/a	Confirmed	
☐	☒	The exact sample size (*n*) for each experimental group/condition, given as a discrete number and unit of measurement
☐	☒	A statement on whether measurements were taken from distinct samples or whether the same sample was measured repeatedly
☐	☒	The statistical test(s) used AND whether they are one- or two-sided<br>*Only common tests should be described solely by name; describe more complex techniques in the Methods section.*
☒	☐	A description of all covariates tested
☐	☒	A description of any assumptions or corrections, such as tests of normality and adjustment for multiple comparisons
☐	☒	A full description of the statistical parameters including central tendency (e.g. means) or other basic estimates (e.g. regression coefficient) AND variation (e.g. standard deviation) or associated estimates of uncertainty (e.g. confidence intervals)
☐	☒	For null hypothesis testing, the test statistic (e.g. *F*, *t*, *r*) with confidence intervals, effect sizes, degrees of freedom and *P* value noted<br>*Give P values as exact values whenever suitable.*
☒	☐	For Bayesian analysis, information on the choice of priors and Markov chain Monte Carlo settings
☒	☐	For hierarchical and complex designs, identification of the appropriate level for tests and full reporting of outcomes
☐	☒	Estimates of effect sizes (e.g. Cohen's *d*, Pearson's *r*), indicating how they were calculated

Our web collection on statistics for biologists contains articles on many of the points above.

Software and code

Policy information about availability of computer code

Data collection	No software was used.
Data analysis	Statistical analyses were performed using GraphPad Prism version 9.3.1 (GraphPad) and SPSS version 24 (IBM).

For manuscripts utilizing custom algorithms or software that are central to the research but not yet described in published literature, software must be made available to editors and reviewers. We strongly encourage code deposition in a community repository (e.g. GitHub). See the Nature Portfolio guidelines for submitting code & software for further information.

Data

Policy information about availability of data

All manuscripts must include a data availability statement. This statement should provide the following information, where applicable:

- Accession codes, unique identifiers, or web links for publicly available datasets
- A description of any restrictions on data availability
- For clinical datasets or third party data, please ensure that the statement adheres to our policy

Restrictions apply to the availability of ctDNA sequences in the national cohort which were obtained from Guardant Health due to data privacy regulations and restrictions for use in the patient consent form. These may be potentially accessed via a collaborative research agreement with Guardant Health. All remaining relevant data are available in the article, Supplementary Information, and source data provided with this paper.

Human research participants

Policy information about studies involving human research participants and Sex and Gender in Research.

Reporting on sex and gender	No gender or sex-based analyses were performed.
Population characteristics	De-identified patient data for the national cohort was provided by the data sponsor (Guardant Health). All patients were required to have advanced non-colorectal gastrointestinal cancers undergoing systemic therapy. Patients in the UTSW/PHS cohort were required to have non-colorectal gastrointestinal cancers treated with systemic therapy and had serial ctDNA testing irrespective of age, sex, or race. Average age in the UTSW/PHS cohort was 66.5.
Recruitment	No recruitment was performed.
Ethics oversight	This retrospective cohort analysis was conducted after receiving institutional review board approval (IRB) at the University of Texas Southwestern Medical Center (UTSW).

Note that full information on the approval of the study protocol must also be provided in the manuscript.

Field-specific reporting

Please select the one below that is the best fit for your research. If you are not sure, read the appropriate sections before making your selection.

- [x] Life sciences
- [ ] Behavioural & social sciences
- [ ] Ecological, evolutionary & environmental sciences

For a reference copy of the document with all sections, see nature.com/documents/nr-reporting-summary-flat.pdf

Life sciences study design

All studies must disclose on these points even when the disclosure is negative.

Sample size	No sample size was predetermined as this analysis was exploratory. Our cohorts were inclusive of all patients in the study time period with ctDNA profiling (n=613 patients in National cohort and n=379 patients in the UTSW cohort). These sample sizes were considered sufficient for capturing prevalent molecular alterations across cancer types.
Data exclusions	No patients were excluded.
Replication	Our study is descriptive in nature and is the first to assess ctDNA changes over time for non-colorectal gastrointestinal cancers in a large-scale and systematic fashion. Therefore, replication is not possible due to the lack of similar datasets.
Randomization	No randomization was used in our analysis as this was a retrospective cohort study. Adjusting for covariates was not performed as the primary aims were to describe genetic features and actionability of ctDNA profiles.
Blinding	No blinding was used in our analysis as this was a retrospective cohort study.

Reporting for specific materials, systems and methods

We require information from authors about some types of materials, experimental systems and methods used in many studies. Here, indicate whether each material, system or method listed is relevant to your study. If you are not sure if a list item applies to your research, read the appropriate section before selecting a response.

Materials & experimental systems

n/a	Involved in the study	
[x]	[ ]	Antibodies
[x]	[ ]	Eukaryotic cell lines
[x]	[ ]	Palaeontology and archaeology
[x]	[ ]	Animals and other organisms
[x]	[ ]	Clinical data
[x]	[ ]	Dual use research of concern

Methods

n/a	Involved in the study	
[x]	[ ]	ChIP-seq
[x]	[ ]	Flow cytometry
[x]	[ ]	MRI-based neuroimaging
